# Cognitive profile and inflammatory markers in children and adolescents with specific learning disorder: a cross-sectional study

**DOI:** 10.3389/fped.2026.1757441

**Published:** 2026-04-13

**Authors:** Umut Balatacı, Bari Ay

**Affiliations:** 1 Private Practitioner, Child and Adolescent Psychiatry, İstanbul, Türkiye; 2 Private Practitioner, Clinic of Child and Adolescent Psychiatry, Private Clinic, Karabük, Türkiye

**Keywords:** healthy controls, inflammation, specific learning disorder, vitamin B12, WISC-IV

## Abstract

**Background:**

Specific learning disorder (SLD) is frequently characterized by weaknesses in working memory and processing speed; however, its biological correlates remain unclear. In this study, we investigate whether inexpensive, blood count–derived inflammation composites and micronutrients are related to cognitive variation in SLD.

**Methods:**

In this cross-sectional study, participants in the SLD group underwent Wechsler Intelligence Scale for Children–Fourth Edition assessment to characterize their cognitive profile, yielding a Full-Scale IQ (FSIQ) and four index scores: Verbal Comprehension, Perceptual Reasoning, Working Memory, and Processing Speed. Fasting blood samples were obtained from all participants for routine hematology/biochemistry, vitamin B12 and folate, C-reactive protein (CRP), and the composite indices, the systemic immune-inflammation index (SII), the systemic inflammation response index (SIRI), and the pan-immune-inflammation value (PIV).

**Results:**

We enrolled 52 children/adolescents with SLD and 56 age-matched healthy controls (HC). The groups were similar in terms of age and sex. Compared with the HC group, the SLD group had a lower vitamin B12 (*p* = 0.008) and a higher PIV (*p* = 0.025), whereas CRP, SII, SIRI, and folate levels did not differ significantly. Within the SLD group, higher cell-based inflammatory indices correlated with poorer cognitive performance, most consistently for the PIV in relation to processing speed and working memory, while vitamin B12 correlated positively with FSIQ, working memory, and processing speed. In the adjusted analyses of the full case–control sample, a higher PIV was independently associated with an increased likelihood of SLD, whereas a higher vitamin B12 level was independently associated with a decreased likelihood of SLD. SII and SIRI exhibited positive trend-level associations, whereas folate showed no association with group membership.

**Conclusions:**

In this case–control study, SLD was associated with lower vitamin B12 levels and higher PIV values than healthy controls. Within the SLD group, higher inflammatory composite indices and lower vitamin B12 levels were associated with poorer cognitive performance, particularly in processing speed and working memory. These findings should be interpreted as associations within a case–control framework and warrant further longitudinal and mechanistic studies.

## Introduction

Specific learning disorder (SLD) is a DSM-5-defined neurodevelopmental disorder characterized by persistent difficulties in learning and using academic skills, including reading, written expression, and/or mathematics. According to Diagnostic and Statistical Manual of Mental Disorders, Fifth Edition (DSM-5), a diagnosis requires that these difficulties persist for at least 6 months despite targeted intervention. Furthermore, the affected academic skills must be substantially below those expected for age and must interfere with academic or daily functioning. The onset of these difficulties should occur during the school-age years, and they should not be better explained by intellectual disability, uncorrected sensory problems, neurological disease, psychosocial adversity, or inadequate instruction (1).

These difficulties emerge during the development of, and reflect atypical maturation of, distributed neural systems that support language, executive control, and efficient information processing. SLD is common in school-age populations and frequently co-occurs with other neurodevelopmental conditions such as Attention-Deficit/Hyperactivity Disorder (ADHD), underscoring its public health and educational importance (2). Current models view SLD as a heterogeneous neurodevelopmental condition shaped by multiple interacting risk factors, including genetic, cognitive, language-related, and environmental influences. Consequently, affected children may exhibit different patterns of reading, writing, or mathematics difficulties, with varying cognitive and psychiatric comorbidities (3).

Wechsler Intelligence Scale for Children–Fourth Edition (WISC-IV)-based studies have reported that children with SLD often show relative weaknesses in the Working Memory Index (WMI) and Processing Speed Index (PSI) compared with the Verbal Comprehension Index (VCI) and Perceptual/Practical Reasoning Index (PRI), although this pattern is not uniform across all cases. Large samples and subgroup analyses across reading, writing, and mathematics presentations have supported this trend, while also emphasizing heterogeneity across SLD profiles and cautioning against overinterpreting individual cognitive patterns (4, 5). Although working memory and processing speed weaknesses are commonly reported in SLD, they represent only part of the broader clinical presentation of the disorder, which may include differing profiles of reading, writing, and mathematics difficulties, as well as language-related impairments that are central to the diagnosis (4, 5).

Converging evidence suggests that immune-inflammatory mechanisms may modulate neurodevelopment and cognition processes. Even low-grade systemic inflammation may influence the developing brain through cytokine-mediated signaling and microglial activation, with downstream effects on oligodendrocyte maturation and myelination, potentially compromising white matter integrity and neural transmission efficiency, processes directly relevant to processing speed and working memory performance (6).

Experimental and longitudinal developmental studies have indicated that early-life inflammatory burden can be associated with altered brain connectivity and poorer later cognitive outcomes **(**6, 7). Composite hematologic indices derived from routine blood counts—such as the Systemic Immune-Inflammation Index (SII), the Systemic Inflammation Response Index (SIRI), and the Pan-Immune-Inflammation Value (PIV)—capture leukocyte–platelet dynamics and have been associated with cognitive outcomes and pediatric conditions (8).

The PIV integrates neutrophil, platelet, and monocyte counts relative to lymphocytes, aiming to reflect broader systemic immune-inflammatory tone than single ratios **(**9). Recent population-based studies have linked SII/SIRI with cognitive performance; pediatric studies have reported alterations of these markers in neurodevelopmental cohorts; and the PIV has shown diagnostic utility in children with acute inflammatory states, supporting its feasibility in pediatric research (5).

Nonetheless, data directly examining these indices in SLD remain sparse (5, 10–12), and emerging disorder-focused evidence suggests that complete blood count (CBC)-derived inflammatory indices may be altered in children with SLD compared with healthy peers **(**9). C-reactive protein (CRP) was included as a conventional inflammatory benchmark to allow comparison between a standard acute-phase marker and the composite blood count–derived indices (SII, SIRI, and PIV), which may better reflect the low-grade systemic immune-inflammatory tone (13).

Micronutrient status represents another biologically plausible pathway. Vitamin B12 and folate are essential for neurodevelopment, myelination, and one-carbon metabolism, and insufficiency of these nutrients has been associated with adverse cognitive and neurobehavioral outcomes in children. While most previous research has focused on early development or other neurodevelopmental conditions, their role in SLD has not been systematically characterized in conjunction with inflammatory indices (14–16).

Because direct SLD-specific evidence on blood-based inflammatory markers remains limited, the role of vitamin B12 and folate in SLD has not been well characterized, and few studies have jointly examined biological markers together with cognitive profiles within SLD, the present cross-sectional case–control study was designed to (i) compare routine laboratory/inflammatory markers (including SII, SIRI, PIV, CRP, vitamin B12, and folate) between children/adolescents with SLD and healthy controls (HCs); (ii) characterize WISC-IV cognitive indices within the SLD group and evaluate correlations between cognitive indices and biological measures; and (iii) examine, in the full case–control sample, which biological variables were independently associated with diagnostic group status (SLD vs. healthy control). By integrating standardized cognitive assessment with composite immune-inflammatory indices and vitamin status, this study aims to clarify the potential biological correlates of the cognitive presentation of SLD and inform future mechanistic and interventional research.

Finally, emerging pediatric literature has begun to implicate low-grade inflammation in SLD specifically (17), but comprehensive models that align WISC-IV profiles with multiparameter inflammatory composites and micronutrients are still limited—highlighting the novelty and relevance of the current investigation.

## Methods

### Study design and setting

The study protocol was reviewed and approved by the Karabük University Non-Interventional Clinical Research Ethics Committee (Decision no. 2025/2420; 28 July 2025). Written informed consent was obtained from the parents/guardians and assent from the children in accordance with the Declaration of Helsinki. The study involved (i) a case–control comparison of laboratory markers (SLD vs. healthy controls) and (ii) within-group cognitive profiling and biomarker–cognition correlation analyses restricted to the SLD group. Participants were recruited from the Child and Adolescent Psychiatry outpatient clinic of a university-affiliated training and research hospital in Karabük, Türkiye, and from routine well-child visits/community sources for the HC group.

Two groups were enrolled: children/adolescents diagnosed with SLD according to DSM-5 and age-comparable HCs. The inclusion criteria for both groups were age 7–16 years, native or fluent Turkish, ability to complete the study procedures, and written parental consent with child assent when applicable. Additional inclusion criteria for the SLD group were at least 1 year of formal schooling, a DSM-5-based clinical diagnosis of SLD supported by documentation of academic underachievement, and a Full-Scale IQ (FSIQ) (WISC-IV) ≥ 80 to exclude intellectual disability. For HCs, additional criteria were typical developmental and educational history, no school-reported learning difficulties, no history of special education services, and no lifetime history of psychiatric, neurodevelopmental, or learning disorders.

The exclusion criteria for both groups included neurological disorders affecting cognition, genetic syndromes, autism spectrum disorder, psychotic or bipolar disorders, major depressive or anxiety disorders requiring pharmacotherapy, current substance use, major uncorrected sensory deficits interfering with testing, and recent use of medications known to affect cognition or inflammation. To ensure interpretable laboratory indices, participants with acute infection, recent vaccination, chronic inflammatory/autoimmune, hematologic, renal, hepatic, or endocrine disease, clinically significant anemia, malignancy, recent surgery or trauma, or recent vitamin B12/folate supplementation were excluded. Fasting blood sampling and correction of routine vision/hearing with the use of aids were permitted.

**Figure 1:**
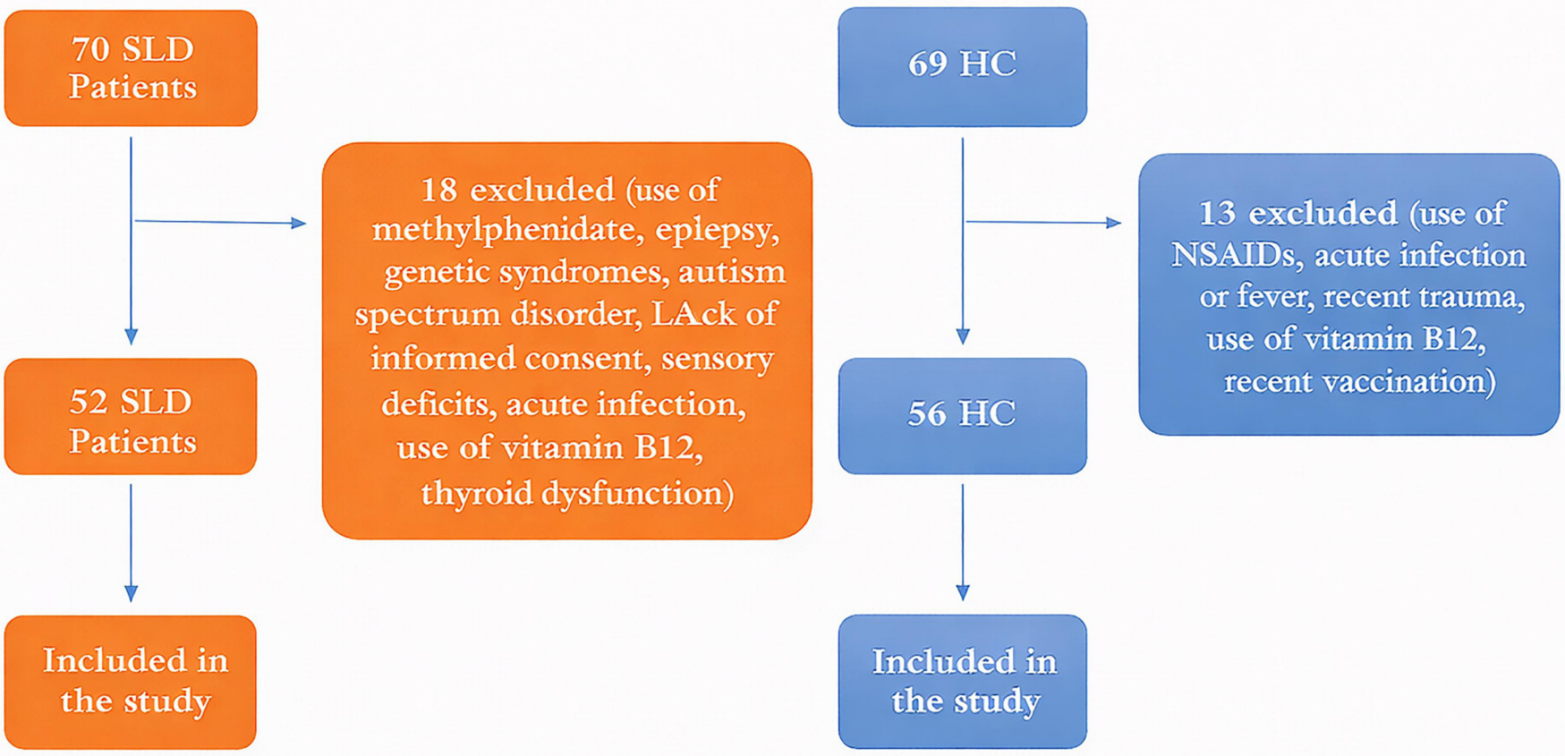
Flowchart of the study.

Psychiatric diagnoses in both groups were established by a child and adolescent psychiatrist using DSM-5-based clinical evaluations and reviews of available medical and school records. In the SLD group, comorbid neurodevelopmental and psychiatric conditions were assessed during the same evaluation, and participants with pharmacologically treated or clinically impairing ADHD were excluded to minimize the confounding effect of inflammatory measures. Because a standardized ADHD symptom rating scale was not uniformly administered, residual confounding related to subthreshold ADHD symptoms could not be entirely ruled out. A flowchart of the study is presented in Figure 1.

### Cognitive assessment

To characterize the cognitive profile of SLD, participants in the SLD group were assessed using the WISC-IV, a psychologist-administered instrument for ages 6:0–16:11 that provides a FSIQ and four index scores: VCI, PRI, WMI, and PSI. The Turkish version (WÇZÖ-IV) was adapted and standardized under a TÜBİTAK-supported project, with norms derived from a nationally representative sample. Previous studies have supported its validity and clinical utility in Turkish populations (18–20). In the present study, WISC-IV was administered only to the SLD group because the cognitive component of the study was designed to characterize the neurocognitive profile of clinically diagnosed SLD cases and to examine within-group associations between WISC-IV indices and biological measures. The healthy control group was included primarily as a comparator for laboratory and inflammatory parameters, rather than for formal neurocognitive profiling. Accordingly, between-group analyses were limited to laboratory variables, and cognitive analyses were restricted to the SLD group. Because FSIQ ≥80 was required for SLD group inclusion to exclude intellectual disability, these cognitive findings should be interpreted as secondary correlational analyses within a clinically defined sample.

### Blood sampling and laboratory measures

Fasting venous blood samples were obtained in the morning during routine clinical evaluations. Complete blood counts were performed using a Mindray BC-600 analyzer (Shenzhen Mindray Bio-Medical Electronics Co., Ltd., Shenzhen, China). Serum biochemistry was measured using an Atellica system (Siemens Healthineers, Erlangen, Germany). Hormone assays were performed on an ADVIA Centaur XPT platform (Siemens Healthineers, Erlangen, Germany). Standard hematologic and biochemical parameters included the following: hemoglobin (Hgb), total white blood cell count (WBC), neutrophils, lymphocytes, monocytes, eosinophils, basophils, platelets, glucose, urea, creatinine, aspartate aminotransferase (AST), alanine aminotransferase (ALT), albumin, thyroid function tests (TSH, free T4), CRP, and serum vitamin B12 and folate concentrations, reported in hospital laboratory units. Except for clinically significant anemia, which was screened using age- and sex-specific reference ranges, the laboratory parameters measured were retained in their continuous form for analysis and were not dichotomized according to parameter-specific reference cutoffs. CRP was included as a conventional inflammatory benchmark to contextualize the findings from the composite blood count–derived indices (SII, SIRI, and PIV).

From complete blood counts, we derived composite inflammatory indices using the following conventional formulae:

SII=platelets×neutrophils/lymphocytes

SIRI=neutrophils×monocytes/lymphocytes

PIV=platelets×neutrophils×monocytes/lymphocytes

The primary comparisons were between the SLD and HC groups for laboratory markers (including CRP, SII, SIRI, PIV, vitamin B12, and folate). Within the SLD group, secondary analyses were performed to examine the correlations between the WISC-IV indices (FSIQ, VCI, PRI, WMI, and PSI) and the inflammatory/micronutrient measures.

### Statistical analysis

Analyses were performed using SPSS Mac version 26.0 (IBM Corp., Armonk, NY, USA). Data were inspected for distributional assumptions. Continuous variables were presented as mean ± SD or median (IQR) where appropriate and categorical variables were presented as counts (%). Group comparisons were performed using independent-samples t-tests or Mann–Whitney U tests for continuous variables and *χ*^2^ or Fisher's exact tests for categorical variables. Within the SLD group, correlations between the WISC-IV indices and the laboratory measures (CRP, SII, SIRI, PIV, vitamin B12, and folate) were assessed using Spearman's method. No formal correction for multiple comparisons was applied to these correlation analyses, which were considered exploratory. To examine the factors associated with diagnostic group membership, we performed a binary logistic regression analysis in the full study sample (52 SLD cases and 56 healthy controls), with group status as the dependent variable (SLD = 1, healthy control = 0). Age, sex, SII, SIRI, PIV, vitamin B12, and folate were entered as independent variables to estimate the adjusted odds ratios [Exp(B)] with 95% confidence intervals. The model fit was evaluated using the Hosmer–Lemeshow goodness-of-fit test and Nagelkerke's R^2^. The Hosmer–Lemeshow test indicated adequate model fit (*p* > 0.05), and Nagelkerke’s R^2^ suggested acceptable explanatory power. Multicollinearity was assessed using variance inflation factors (VIF) and tolerance values. All VIF values were below 2, and tolerance values were above 0.5, indicating no evidence of problematic multicollinearity among the independent variables. A two-sided *p* < 0.05 was considered statistically significant.

## Results

### Between-group comparisons

We enrolled 52 children/adolescents with SLD and 56 healthy controls in this study. The groups were comparable in terms of age and sex (Table 1). Most hematologic and biochemical parameters were similar between groups; however, the SLD group had significantly lower vitamin B12 levels and higher PIV values than the healthy controls (Table 2).

**Table 1:** Baseline demographic characteristics of the study groups.

Variable	SLD (*n* = 52)	HC (*n* = 56)	*p*
Age, years	11.65 ± 2.65	11.50 ± 3.28	0.856
Sex, *n* (%)	Male: 32 (61.5%) Female: 20 (38.5%)	Male: 28 (50%) Female: 28 (50%)	0.383
FSIQ	89.24 ± 9.10		
VCI	68.75 ± 18.09		
PRI	76.25 ± 14.19		
WMI	68.06 ± 11.72		
PSI	79.00 ± 12.53		

SLD, specific learning disorder; HC, healthy controls; FSIQ, Full-Scale IQ; VCI, Verbal Comprehension Index; PRI, Perceptual Reasoning Index; WMI, Working Memory Index; PSI, Processing Speed Index.

Data are mean ± SD or *n* (%).

**Table 2:** Laboratory parameters in SLD vs. healthy controls.

Variable	SLD (*n* = 52)	HC (*n* = 56)	*p*
Hgb, g/dL	12.91 ± 1.27	13.36 ± 1.11	0.200
WBC, 10^9^/L	7.92 ± 2.26	6.98 ± 1.58	0.437
Platelets, 10^9^/L	319.00 ± 82.75	274.69 ± 73.32	0.104
Neutrophils, 10^9^/L	4.56 ± 1.93	3.78 ± 1.00	0.084
Lymphocytes, 10^9^/L	2.50 ± 0.71	2.39 ± 0.85	0.629
Monocytes, 10^9^/L	0.55 ± 0.16	0.48 ± 0.14	0.123
Basophils, 10^9^/L	0.1 (0.1–0.1)	0.1 (0–0.1)	0.786
Eosinophils, 10^9^/L	0.15 (0.11–0.23)	0.19 (0.14–0.31)	0.358
Glucose, mg/dL	89.65 ± 9.75	89.53 ± 9.04	0.962
Urea, mg/dL	25.62 ± 5.88	25.42 ± 8.98	0.926
Creatinine, mg/dL	0.59 ± 0.16	0.61 ± 0.18	0.718
AST, U/L	22.35 ± 6.50	23.58 ± 5.63	0.477
ALT, U/L	18.50 ± 7.98	16.23 ± 5.44	0.248
TSH, µIU/mL	2.27 (1.76–4.23)	2.02 (1.46–3.33)	0.297
fT4, ng/dL	1.14 ± 0.22	1.14 ± 0.11	0.990
Albumin, g/dL	4.82 ± 0.34	4.77 ± 0.21	0.578
Vitamin B12, pg/mL	270 (243–329)	346 (290–434)	**0** **.** **008**
Folate, ng/mL	8.31 ± 3.06	8.36 ± 2.23	0.960
CRP, mg/L	1.20 (0.55–7.85)	1.00 (0.50–2.20)	0.322
SII	430.67 (314.16–841.22)	451.36 (317.63–589.65)	0.288
SIRI	0.91 (0.49–1.80)	0.68 (0.53–0.97)	0.439
PIV	266.88 (137.29–532.95)	215.90 (131.31–309.29)	**0**.**025**

SLD, specific learning disorder; HC, healthy controls; Hgb, hemoglobin; WBC, white blood cells; AST, aspartate aminotransferase; ALT, alanine aminotransferase; TSH, thyroid-stimulating hormone; fT4, free thyroxine; SII, systemic immune-inflammation index; SIRI, Systemic Inflammation Response Index; PIV, pan-immune-inflammation value; SD, standard deviation; IQR, interquartile range.

Data are mean ± SD or median (25th–75th percentile).

Bold values indicate statistically significant results (*p* < 0.05).

### Within-SLD analyses

WISC-IV was administered only to the SLD group. Within this subgroup, higher cell-based inflammatory indices were generally associated with poorer cognitive performance, with the most consistent pattern observed for the PIV. The strongest associations involved processing speed and working memory (Table 3).

**Table 3:** Correlations between WISC-IV indices and biological measures within the SLD group.

WISC-IV indices	SII	SIRI	PIV	CRP	Vitamin B12	Folate
FSIQ	r = −0.242 *p* = 0.027	r = −0.220 *p* = 0.062	r = −0.262 *p* = 0.018	r = −0.127 *p* = 0.290	r = 0.222 *p* = 0.041	r = 0.099 *p* = 0.440
VCI	r = −0.124 *p* = 0.280	r = −0.092 *p* = 0.410	r = −0.152 *p* = 0.180	r = −0.072 *p* = 0.520	r = 0.140 *p* = 0.210	r = 0.081 *p* = 0.480
PRI	r = −0.215 *p* = 0.049	r = −0.184 *p* = 0.090	r = −0.237 *p* = 0.035	r = −0.101 *p* = 0.360	r = 0.198 *p* = 0.071	r = 0.052 *p* = 0.660
WMI	r = −0.286 *p* = 0.011	r = −0.245 *p* = 0.028	**r** **=** **−0.308** ***p*** **=** **0.007**	r = −0.165 *p* = 0.130	r = 0.262 *p* = 0.019	r = 0.102 *p* = 0.370
PSI	**r** **=** **−0.312** ***p*** **=** **0.006**	r = −0.273 *p* = 0.014	**r** **=** **−0.341** ***p*** **=** **0.003**	r = −0.186 *p* = 0.097	**r** **=** **0.290** ***p*** **=** **0.009**	r = 0.124 *p* = 0.290

WISC-4, Wechsler Intelligence Scale for Children–Fourth Edition; FSIQ, Full-Scale IQ; VCI, Verbal Comprehension Index; PRI, Perceptual Reasoning Index; WMI, Working Memory Index; PSI, Processing Speed Index; SII, Systemic Immune-Inflammation Index; SIRI, Systemic Inflammation Response Index; PIV, pan-immune-inflammation value; CRP, C-reactive protein.

The Spearman correlation test was used.

Bold values indicate statistically significant results (*p* < 0.05).

### Full-sample adjusted model

In a binary logistic regression model including all participants, a higher PIV was independently associated with increased odds of SLD, whereas higher vitamin B12 levels were associated with decreased odds of SLD after adjusting for age and sex (Table 4).

**Table 4:** Binary logistic regression analysis of factors associated with case–control group membership (SLD vs. healthy control).

Variable	B	SE	Wald	*p*	Exp(B)	95% CI for EXP(B) lower	95% CI for EXP(B) upper
Constant	1.420	1.210	1.378	0.240	—	—	—
Age	−0.020	0.058	0.122	0.727	0.980	0.870	1.120
Gender	−0.300	0.360	0.694	0.405	0.741	0.366	1.497
SII	0.003	0.0013	0.884	0.073	1.003	1.001	1.006
SIRI	0.058	0.028	0.725	0.108	1.060	1.003	1.120
PIV	0.246	0.100	6.049	**0** **.** **014**	1.279	1.050	1.490
Vitamin B12	−0.385	0.150	6.594	**0**.**010**	0.680	0.490	0.940
Folate	−0.020	0.031	0.433	0.510	0.980	0.921	1.042

SLD, specific learning disorder; SII, Systemic Immune-Inflammation Index; SIRI, Systemic Inflammation Response Index; PIV, pan-immune-inflammation value.

A binary logistic regression analysis was performed in the full study sample, with group status as the dependent variable (SLD = 1, healthy control = 0).

Bold values indicate statistically significant results (*p* < 0.05).

## Discussion

In the case–control component of this study (laboratory comparisons), children/adolescents with SLD showed significantly lower serum vitamin B12 levels and higher PIV values than healthy controls, whereas CRP, SII, SIRI, and folate levels did not differ significantly between the groups. Within the SLD group, higher cell-based inflammatory composites—most consistently the PIV—were associated with poorer cognitive performance, particularly in terms of processing speed and working memory. In the adjusted analyses of the full case–control sample, a higher PIV was independently associated with an increased likelihood of SLD, while higher vitamin B12 levels were associated with a decreased likelihood of SLD.

An additional point requiring caution is the interpretation of the PIV. Although a higher PIV was associated with case–control group membership in our sample, this finding should not be interpreted as evidence that the PIV is specific to SLD. Our comparison was limited to children/adolescents with SLD and healthy controls; we did not include other clinically referred neurodevelopmental or psychiatric groups. Therefore, an elevated PIV may reflect a broader correlation with clinical presentation or low-grade systemic immune activation rather than a disorder-specific feature of SLD. Future studies, including both healthy controls and non-SLD clinical comparison groups, are required to determine the specificity and incremental diagnostic value of the PIV.

In our clinically referred SLD sample, WISC-IV index scores were globally low, with relatively higher PRI and PSI compared with VCI and WMI. This profile is compatible with an efficiency-based vulnerability (working memory/processing efficiency) often reported in SLD, while the very low mean FSIQ underscores that our findings may reflect a more impaired, treatment-seeking subgroup and may not generalize to higher-functioning SLD populations.

Our findings extend the characterization of SLD by linking its cognitive vulnerabilities—especially slower processing speed and weaker working memory—to two accessible biological signals: higher cell-based inflammatory composites (most robustly PIV) and lower levels of vitamin B12. This direction is consistent with disorder-specific pediatric evidence: Avşar et al. reported higher CBC-derived inflammatory markers (including SII and neutrophil-to-lymphocyte ratio (NLR)/platelet-to-lymphocyte ratio (PLR)) in children with SLD compared with controls, and their multivariable analyses suggested an independent association for SII (8). Other SLD-focused studies have reported immune-related alterations (e.g., galectin-1/galectin-3) in treatment-naive children with SLD, supporting a plausible low-grade inflammatory/neuroimmune component in at least a subset of cases (21). At the same time, stronger evidence for links between inflammation and cognition comes largely from broader developmental or adolescent samples rather than from SLD itself (22). Recent work has associated a higher inflammatory burden with poorer neurobehavioral performance in adolescents, and prenatal maternal inflammation has also been linked to less favorable later cognitive outcomes (23). Although these results provide valuable case–control data for an emerging field, inflammatory composites should not yet be regarded as established or specific biomarkers for SLD.

Evidence from population cohorts bolsters the plausibility that composite leukocyte–platelet indices are related to cognition (5, 24). Analyses of US National Health and Nutrition Examination Survey (NHANES) data report that abnormal or higher SII is associated with poorer cognitive performance and, in some studies, interacts with metabolic risks. Complementary analyses identify graded relationships of SII/SIRI with low cognition, consistent with our observation that composites may outperform CRP in capturing cognition-relevant immune tone (5, 17). The PIV, the most integrative of these markers, was originally introduced in oncology and has repeatedly shown prognostic value across diseases, supporting its interpretability as a “pan-immune” index. Its independent association with SLD in our model suggests a potentially relevant signal warranting further study in neurodevelopmental contexts, rather than a specific or clinically established marker (25, 26).

Within the SLD group, higher cell-based inflammatory composites were generally associated with poorer WISC-IV performance, whereas higher vitamin B12 levels were associated with better cognitive scores; no meaningful association was found between folate and the cognitive indices examined. Across indices, SII, SIRI, and especially PIV, correlated negatively with WISC-IV outcomes, with the strongest effects on processing speed and working memory. This domain-specific pattern is biologically plausible, given that processing speed and working memory depend heavily on distributed network efficiency and white matter integrity (27). Neurodevelopmental and neuroimaging literature links processing efficiency to myelination/white matter microstructure in childhood and adolescence (28). Although working memory and processing speed were the most salient WISC-IV-related findings in our sample, these domains should not be interpreted as exhausting the broader cognitive phenotype of SLD, which also includes important language-related and academic skill impairments that are central to clinical diagnosis.

Multivariable logistic regression showed that a higher PIV was independently associated with increased odds of SLD, whereas higher vitamin B12 levels were associated with reduced odds of the disorder relative to healthy controls. The trend-level signals for SII and SIRI suggest that multiple composites may carry overlapping information; however, the PIV appears to provide the most discriminative summary of leukocyte–platelet dynamics in this dataset. Folate was not associated with group membership, and neither age nor sex contributed significantly to the results.

Pediatric reviews and trials link suboptimal B12 levels to poorer cognition, and improving B12 status may benefit neurocognitive outcomes—particularly in populations with low baseline intake (14). Our findings that lower B12 levels characterize the SLD group and that higher B12 levels are associated with better working memory, better processing speed, and lower odds of SLD diagnosis are consistent with the proposed roles of B12 in one-carbon metabolism, myelin integrity, and synaptic efficiency (29–31). Similar caution applies to micronutrient findings. Vitamin B12 is biologically relevant to myelination, one-carbon metabolism, and synaptic function, making its association with working memory and processing speed plausible (32); however, direct SLD-specific evidence remains very limited. Much of the available literature is indirect, originating from broader child cognition studies (32), neurodevelopmental nutrition reviews (14), or related disorders (33), rather than from SLD cohorts. For example, a recent scoping review concluded that evidence linking prenatal vitamin B12 status to children's neurodevelopment remains inconclusive (32), whereas a 2024 systematic review in children with ADHD reported lower vitamin B9/B12 levels than in healthy controls (33). In this context, our finding that lower vitamin B12, but not folate, is associated with SLD and poorer cognitive performance is exploratory and requires further validation. Future studies should determine whether this signal replicates in independent SLD samples and whether more functionally informative markers of B12 status, such as homocysteine or methylmalonic acid, add explanatory value beyond serum B12 levels.

CRP levels did not differ between groups and were not correlated with cognition in our sample. This finding should be interpreted cautiously, because the CRP values observed were low and not suggestive of clinically overt inflammatory states. As a conventional acute-phase reactant, CRP may be less informative in the context of subtle or low-grade immune variation than cell-based composite indices, which may better capture the distributed hematologic inflammatory tone (13). Folate also showed no meaningful associations in our sample.

Although clinically impairing or pharmacologically treated ADHD was excluded in the present study, ADHD remains highly relevant when interpreting the specificity of inflammatory findings in SLD because it is a common comorbidity, and subthreshold symptoms could not be fully quantified in all participants. In addition, previous literature suggests that CBC-derived inflammatory alterations may extend across neurodevelopmental conditions rather than being unique to SLD (34). Accordingly, future studies should test whether PIV/SII/SIRI differentiates SLD-only presentations from SLD with comorbid ADHD and whether observed associations with processing speed and working memory remain after dimensional adjustment for ADHD symptom burden.

## Limitations

Several limitations should be considered when interpreting the findings of this study. First, the cross-sectional, single-center design precludes causal inference, and the modest sample size—together with the absence of an *a priori* sample size calculation due to the retrospective design—limits confidence in null or trend-level findings and increases the possibility of type II error. In addition, the sample size was modest relative to the number of predictors included in the logistic regression model. This may have limited statistical power, particularly for detecting small independent associations, and may have reduced model stability by increasing the risk of overfitting and imprecise coefficient estimates. This study involved multiple correlation analyses between cognitive indices and biological markers, and no formal correction for multiple comparisons was applied. As a result, the possibility of a type I error cannot be excluded, and some nominally significant associations may reflect chance findings. Therefore, the adjusted regression findings should be interpreted cautiously and regarded as exploratory pending replication in larger independent samples. Second, laboratory biomarkers were measured at a single time point, and residual confounding was possible. Important factors that may influence inflammatory markers and micronutrient levels were not systematically assessed, including broader dietary and nutritional status, socioeconomic conditions, BMI or adiposity, sleep patterns, iron deficiency or other subclinical nutritional deficiencies, and recent allergies or infection history. These factors may affect both systemic inflammatory indices and vitamin levels and, therefore, could have influenced the observed associations. Accordingly, the relationships observed between inflammatory markers, vitamin B12, and cognitive measures should be interpreted with caution, as they may partly reflect unmeasured background health, nutritional, or environmental differences between the participants.

Third, the cognitive component of the study was limited by the fact that WISC-IV was administered only to the SLD group. As a result, direct between-group comparisons of cognitive profiles were not possible, and the absence of standardized cognitive testing in healthy controls introduces a risk of differential assessment bias and incomplete cognitive comparability between the groups. In the control group, typical cognitive functioning was inferred from developmental and educational history, absence of school-reported learning difficulties, and the lack of psychiatric or neurodevelopmental diagnoses based on clinical evaluation and exclusion criteria; however, this approach may not have fully excluded subtle cognitive difficulties. Therefore, the observed biomarker–cognition associations should be interpreted as within-SLD findings rather than as evidence of case–control cognitive differences.

Fourth, age- and sex-specific reference ranges were applied only for the exclusion of clinically significant anemia. The remaining laboratory markers were analyzed as continuous variables and were not interpreted according to analyte-specific pediatric reference intervals. Accordingly, the present findings should be understood as between-group differences and within-sample associations rather than as evidence of the prevalence of laboratory abnormalities relative to pediatric normative thresholds. Finally, although clinically impairing or pharmacologically treated ADHD was excluded from the analyzed sample, subthreshold ADHD symptoms were not dimensionally assessed in all participants and, therefore, cannot be fully ruled out as a source of residual confounding. Therefore, the apparent specificity of the observed immune-inflammatory associations with SLD should be interpreted cautiously.

## Conclusions and future directions

Overall, higher PIV and lower vitamin B12 levels were observed in the SLD group relative to healthy controls, and within the SLD group, higher cell-based inflammatory composites—most consistently PIV—were associated with poorer processing speed and working memory. Future studies should pursue longitudinal pediatric designs with repeated immune/micronutrient assessments, detailed diet and sleep measures, and neuroimaging of white matter pathways supporting speed and working memory. Trials targeting nutritional optimization (B12) and, more speculatively, interventions that attenuate low-grade inflammation may help clarify whether these biological pathways are meaningfully related to efficiency-based cognitive domains in SLD. Accordingly, the present findings should be interpreted as associations within a case–control framework rather than as evidence of stand-alone diagnostic or predictive utility or disorder specificity.

## Data Availability

The raw data supporting the conclusions of this article will be made available by the authors without undue reservation.
